# Transcriptomic Profiles of Monocyte-Derived Macrophages in Response to *Escherichia coli* is Associated with the Host Genetics

**DOI:** 10.1038/s41598-019-57089-0

**Published:** 2020-01-14

**Authors:** Mehdi Emam, Angela Cánovas, Alma D. Islas-Trejo, Pablo A. S. Fonseca, Juan. F. Medrano, Bonnie Mallard

**Affiliations:** 1 Department of Pathobiology, Ontario Veterinary College, University of Guelph, Guelph, Ontario Canada; 2 Centre for Genetic Improvement of Livestock, Department of Animal Biosciences, Ontario Agricultural College, University of Guelph, Guelph, Ontario Canada; 3 Department of Animal Science, University of California-Davis, Davis, California USA

**Keywords:** Immunogenetics, Gene expression profiling

## Abstract

Reactive Nitrogen Species (RNS) are a group of bactericidal molecules produced by macrophages in response to pathogens in a process called oxidative burst. Nitric oxide (NO^−^) is a member of RNS produced from arginine by inducible Nitric Oxide Synthase (iNOS) enzyme. The activity of iNOS and production of NO^−^ by macrophages following stimulation is one of the indicators of macrophage polarization towards M1/proinflammatory. Production of NO^−^ by bovine monocyte-derived macrophage (MDM) and mouse peritoneal macrophages has been shown to be strongly associated with host genetic with the heritability of 0.776 in bovine MDM and 0.8 in mouse peritoneal macrophages. However, the mechanism of genetic regulation of macrophage response has remained less explored. In the current study, the transcriptome of bovine MDMs was compared between two extreme phenotypes that had been classified as high and low responder based on NO^−^ production. The results showed that 179 and 392 genes were differentially expressed (DE) between high and low responder groups at 3 and 18 hours after exposure to *Escherichia coli*, respectively. A set of 11 Transcription Factors (TFs) (*STAT1*, *IRF7*, *SPI1*, *STAT4*, *IRF1*, *HIF1A*, *FOXO3*, *REL*, *NFAT5, HIC1*, and *IRF4*) at 3 hours and a set of 13 TFs (*STAT1*, *IRF1*, *HIF1A*, *STAT4*, *ATF4*, *TP63*, *EGR1*, *CDKN2A*, *RBL1, E2F1, PRDM1, GATA3*, and *IRF4*) at 18 hours after exposure to *E. coli* were identified to be differentially regulated between the high and low responder phenotypes. These TFs were found to be divided into two clusters of inflammatory- and hypoxia-related TFs. Functional analysis revealed that some key canonical pathways such as phagocytosis, chemotaxis, antigen presentation, and cell-to-cell signalling are enriched among the over-expressed genes by high responder phenotype. Based on the results of this study, it was inferred that the functional characteristics of bovine MDMs are associated with NO-based classification. Since NO^−^ production is strongly associated with host genetics, this study for the first time shows the distinct proinflammatory profiles of macrophages are controlled by the natural genetic polymorphism in an outbred population. In addition, the results suggest that genetics can be considered as a new dimension in the current model of macrophage polarization which is currently described by the combination of stimulants, only.

## Introduction

The immune system is an intricate network of cells and molecules, providing several layers of protection against pathogens. Approximately, 8,000 genes in human and mouse genomes and 5,500 genes in the bovine genome are annotated with the “immune response” term (Ensembl, release 96)^1^. This notable number of genes represents the complexity of genetic regulation of the immune system. Understanding the genetic regulation of immune system in health and disease can shed light on many gaps in the current knowledge about individual differences between patients in the progress of their diseases and also provide the foundation of precision medicine^2,3^. In livestock, this information can help to breed livestock that is naturally more resistant to diseases and help to reduce the use of antibiotics on farm^4^. However, investigating genetic control of the immune system is remarkably challenging. Besides the number of genes controlling this system, environmental and physiological factors, and the dynamic interactions between the host-pathogens and host-microbiota have been limiting factors to precisely determine the causal mechanism in resistance or susceptibility to disease^4^. One of the primary barriers has been an optimized strategy to measure susceptibility or resistance, particularly in the context of complex disease traits^4,5^. Recently, along with technical advances in cellular biology and genetics, reductionist approaches are gaining more attention to reduce the complexity of the various interactions by investigating a sub-system instead of the whole immune system^3,6,7^. In the reductionist approaches, the interactions of the sub-system with pathogens are examined in a controlled environment. In this approach, the performance of the sub-system is the criterion of classification, instead of the holistic approach that is based on the outcome of infection^8,9^. Due to the fact that the sub-system, i.e. a cellular function, is controlled by a limited number of genes under a controlled environment, the probability of finding causal mechanisms is much higher in comparison to holistic approaches^9,10^.

Among cells and molecules of the immune system, macrophages are the cornerstone of the innate immune system. They are equipped with a wide range of functions from pro-inflammatory (at the beginning of infection) to anti-inflammatory and wound healing (at the end of infection). These cells are among the first responder to infections. Macrophages are present in tissues either before the exposure to pathogens, Tissue-Resident Macrophages (TRM), or they are transformed from monocytes after the exposure to pathogens, Monocyte-Derived Macrophages (MDM)^11–13^. During inflammation, MDMs play a crucial role in eliminating the pathogens, attracting cells of the adaptive immune system to the tissue, and re-stimulating the lymphocytes as they enter into the inflamed tissues^14^. Phagocytosis and production of Reactive Oxygen and Nitrogen Species (ROS and RNS) via the oxidative burst process are the main microbicidal functions of macrophages^15^. Nitric oxide (NO^−^) is a member of the RNS produced by macrophages with strong bactericidal activity^16^. Inducible Nitric Oxide Synthase (iNOS) utilize L-arginine and NADP + to produce NO^−^. However, L-arginine can be used by Arginase 1 (Arg1) to produce polyamines^17^. The magnitude of utilization of L-arginine by iNOS or Arg1 has been documented to be associated with the proinflammatory profile of macrophage, also known as M1 and M2 polarization^18,19^. Although the detailed mechanism of macrophage polarization in regard to stimulation is very well known and the individual variation in cytokine production in human population has been reported, the mechanism of genetic regulation has remained unanswered^20^. Recently, the reductionist approach has been utilized to investigate the effect of host genetics on the *in-vitro* functional variation of bovine MDM in response to *Escherichia coli* (*E. coli*). The study revealed notable individual variation in NO^−^ production and also positive correlation between the phagocytic ability of MDMs and NO^−^ production. Furthermore, the results showed that the genetics of the host determined 77% of the phenotypic variation in NO production^21^. However, it is not known if individual variation in production of NO^−^ in response to one stimulant is associated with overall distinct proinflammatory profiles of macrophages, similar to distinct macrophage inflammatory profile in response to different stimulants. Based on the literature, it was hypothesized that the inflammatory profile of MDMs differs between classes of macrophages ranked based on NO^−^ production^22,23^. In addition, the observed functional variation in the response of MDMs to bacterial treatment is expected to be governed through the differences in the abundance of intracellular signalling components. In the current study, MDMs from cows, that were previously classified into high and low responder groups based on the *in-vitro* NO^−^ response, were stimulated with *E. coli* and the transcriptome of MDMs at two time points were analyzed by RNA-Seq technology. The differentially expressed genes were identified based on the *in-vitro* phenotype, corrected by untreated controls in each time point. Functional analysis was performed to identify metabolic pathways and to predict the biological consequence associated with the phenotypic class of MDMs.

## Results

**Figure 1:**
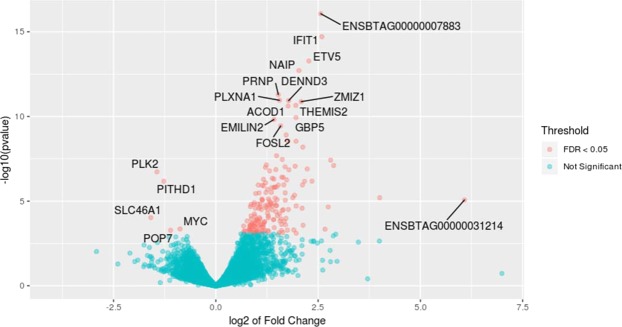
Volcano plot of mRNA expression in monocyte-derived macrophage at 3 hours after treatment with *Escherichia coli*. Fold changes in expression are calculated by deducting the expression value of low responder group (treated samples, n = 3) from high responder group (treated sample, n = 3) and adjusted for untreated groups (n = 3/phenotype) within each phenotype.

At 3 hours after treatment, the average number reads which passed the quality control was 18.1 and 15.6 million reads per sample for the control and treatment group, respectively. In both groups, more than 96% of the reads were uniquely mapped to the bovine reference genome. Comparing the gene expression between the two phenotypic groups, 179 genes were identified with the absolute FC of > 1.5 and the FDR < 0.05 (Fig. 1, Supplementary Table 1). Among these genes, 174 genes were over expressed in the high NO^−^ responder group and 5 genes were more expressed in the low NO^−^ responder group. The average of FC in the top 10 over expressed genes was 8.55 (average DESeq2 base mean of 926.1) and the average of the 5 DE genes more expressed in the low NO^−^ responder group was 2.39 (average DESeq2 base mean of 568.0).

**Figure 2:**
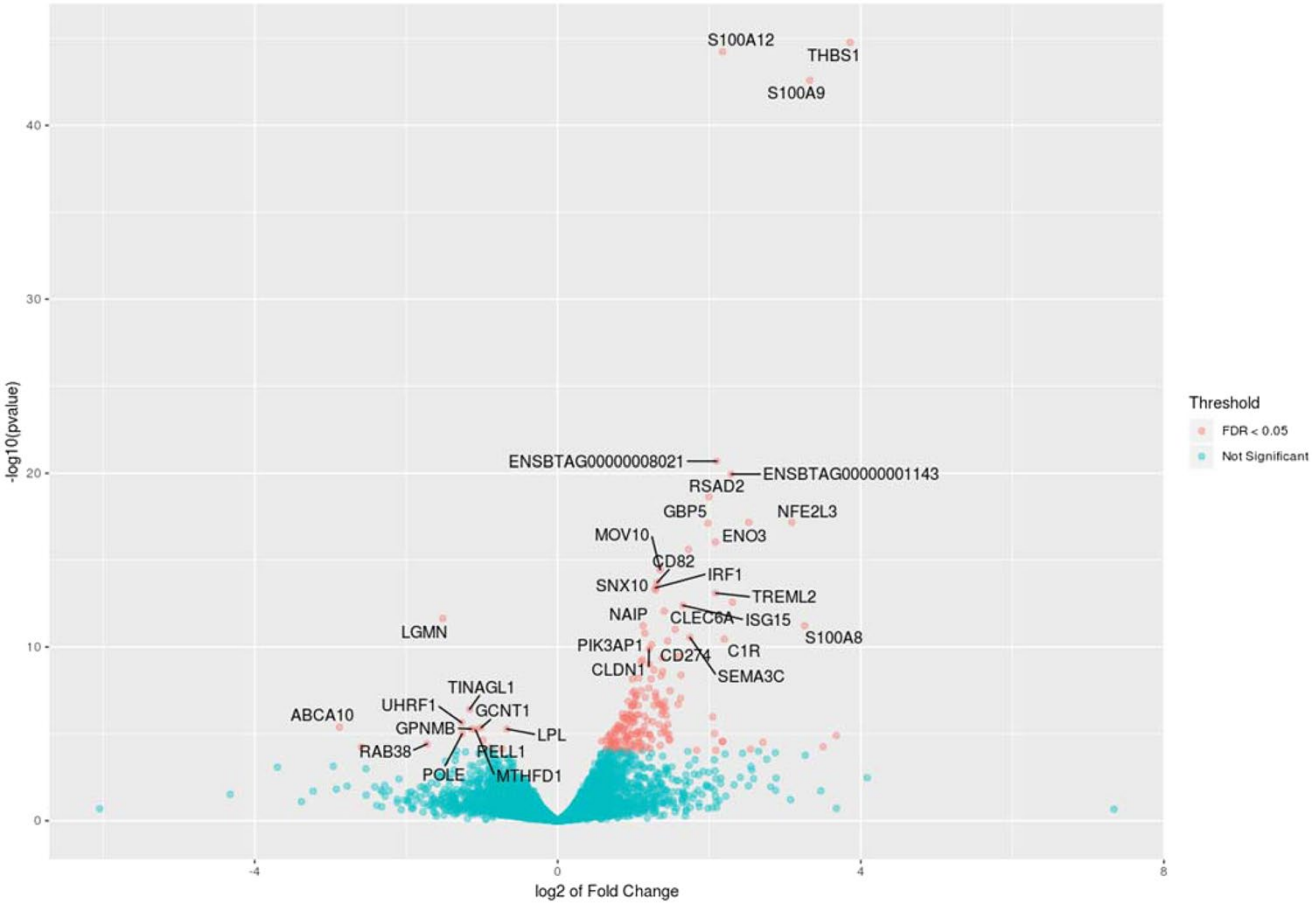
Volcano plot of mRNA expression in monocyte-derived macrophage at 18 hours after treatment with *Escherichia coli*. Fold changes in expression are calculated by deducting the expression value of low responder group (treated samples, n = 3) from high responder group (treated sample, n = 3) and adjusted for untreated groups (n = 3/phenotype) within each phenotype.

**Figure 3:**
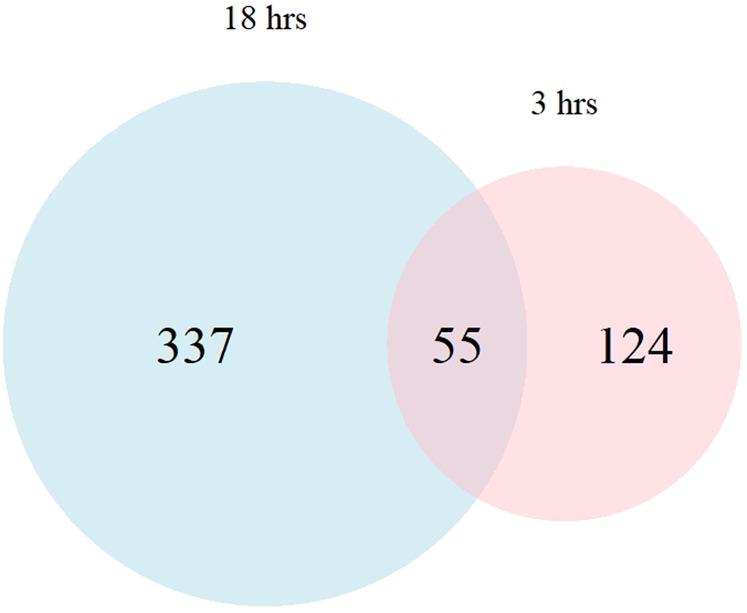
Venn diagram of differentially expressed genes in monocyte-derived macrophages at 3 (pink) and 18 hours (blue) after stimulation with *Escherichia coli*.

At 18 hours after treatment, the average number reads which passed the quality control was 21.5 and 21.7 million reads per sample for the control and treatment group, respectively. In both groups, more than 95% of the reads were uniquely mapped to the bovine reference genome. Comparing the gene expression between the two phenotypic groups, 392 genes were identified with the absolute FC of > 1.5 and the FDR < 0.05 (Fig. 2, Supplementary Table 2). Among these genes, 326 genes were overexpressed in the high NO^−^ responder group and 66 genes were more expressed in the low responder NO^−^ group. The average of FC for the top 10 overexpressed genes was 9.32 (average DESeq2 base mean of 1249.7) and the average of the top 10 more DE expressed genes in the low responder group was 3.59 (average DESeq2 base mean of 2435.7). Among DE genes at both 3 and 18 hours after *E. coli* treatment, 55 genes were in common (Fig. 3).

It is worth emphasizing that the DE genes that were identified in this study are not only corrected against the untreated control, but the FCs in the expression are calculated based on the differences between two groups of genetically distinct MDMs. These two groups were exposed to the same bacteria with exactly similar MOI in a similar environment. The only difference between these two groups was their genetic architecture as we have previously shown^21^.

**Figure 4:**
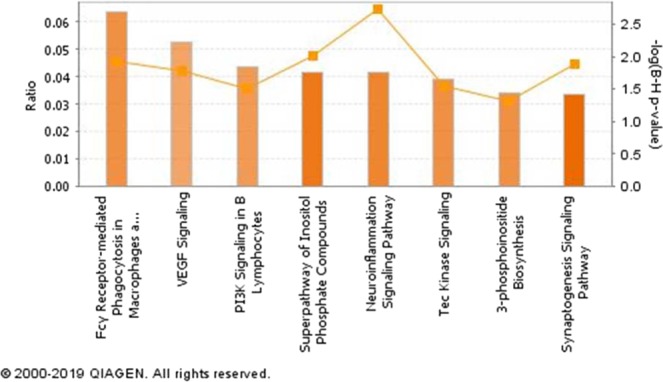
Canonical pathways associated with differentially expressed genes at 3 hours after treatment. The density of orange colour in each column represent the positive activation z-score (all these pathways >2.0). The orange line represents the FDR p-value for each pathway (all pathways <0.05).

**Figure 5:**
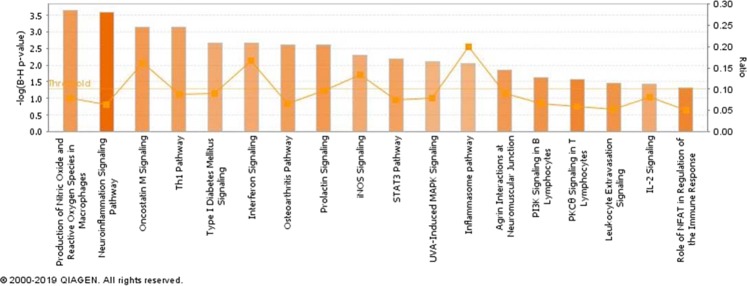
Canonical pathways associated with differentially expressed genes at 18 hours after treatment. The density of orange colour in each column represent the positive activation z-score (all these pathways >2.0). The orange line represents the enrichment ratio for each pathway (all pathways >0.05).

The list of DE genes was analyzed in IPA. In this analysis, FDR of 0.1 and absolute log2FC of 0.58 (equal to 1.5 FC) were set as the criteria to maximize the discovery of the pathways that are associated with different phenotypes. At the first step of the analysis, 8 and 18 canonical pathways were identified with FDR < 0.05 and z score of ≥2, at 3- and 18-hours post-treatment, respectively (Figs. 4 and 5). Of note, FC-γ mediated phagocytosis, and 3-phosphoinositide biosynthesis pathways were identified to be associated with high responder groups at the first time point (3 hours, Fig. 4). At the 18 hours time point, production of NO and reactive oxygen species, Th1 pathway, inflammasome pathway and leukocyte extravasation signalling were among the associated pathways with MDMs phenotypic groups (Fig. 5).

**Figure 6:**
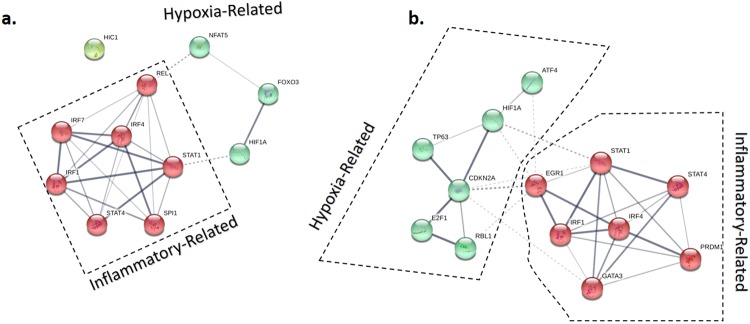
The interaction network among the transcription factors that were differentially regulated between the extreme phonotypes of high and low responder based on production of nitric oxide after 3 (**a**) and 18 (**b**) hours exposure to *Esherishia coli*. The differentially regulated transcription factors were analyzed using the Search Tool for Retrieval of Interacting Genes/Proteins (STRING, v. 11). The inflammatory cluster is represented by the red and the hypoxia-related cluster is represented by green.

All DE genes were screened using IPA to predict the upstream regulators. At both time points, Lipopolysaccharide (LPS) was identified as the most probable upstream regulator of DE genes with the z-score of 9.00 and a p-value of 1e-58 at the second time point (Supplementary Fig. 1). Transcription factors are known to be the master regulators of macrophage functions^24,25^. After applying a filter to only include transcription factors 11 Transcription Factors (TFs) (*STAT1*, *IRF7*, *SPI1*, *STAT4*, *IRF1*, *HIF1A*, *FOXO3*, *REL*, *NFAT5, HIC1*, and *IRF4*) were identified with activation z scores ≥ |2|, p-value between 1.06e-3 to 1.45e-14 and log2FC > |0.58| at the first time point (Supplementary Table 3). At the second time point, another set of 13 TFs (*STAT1*, *IRF1*, *HIF1A*, *STAT4*, *ATF4*, *TP63*, *EGR1*, *CDKN2A*, *RBL1, E2F1, PRDM1, GATA3*, and *IRF4*) were identified with activation z score ≥ |2|, p-value between 3.37e-7 to 1.41e-24 and log2FC ≥ |0.53| (Supplementary Table 4). The interaction analysis revealed that these TFs group in one network, composed of two clusters at each time point. One of the clusters contained TFs with roles in regulation of inflammatory responses and the other cluster contained TFs with roles in response to hypoxia and regulation of the metabolic process of macrophages (Fig. 6).

The list of DE genes was also screen by IPA, to predict cellular process and biological functions that are downstream of DE genes. Among 45 categories of disease and function including 502 different pathways, the top 5 categories based on the proportion of members with z-scores ≥ 2 were identified as “Free Radical Scavenging” (100%, 3 members), “Cell-To-Cell Signalling and Interaction” (53%, 38 members), “Immune Cell Trafficking” (53%, 32 members), Cellular Response” (48%, 41 members), “Inflammatory Response” (29%, 51 members), and “Hematological System Development and Function” (23%, 84 members) at 3 hours post-treatment (Supplementary Fig. 2, Supplementary Table 5). At 18 hours after treatment, the top 5 categories with most members with z-scores ≥ 2 were identified as “Cell-To-Cell Signalling and Interaction” (80%, 60 members), “Immune Cell Trafficking” (78%, 41 members), “Cellular Movement” (68%, 51 members), “Cellular Function and Maintenance” (59%, 39 members), “Hematological System Development and Function” (58%, 96 members). Also, it should be noted that “Inflammatory Response” (48%, 81 members) was the 6^th^ category at 18 hours post-treatment (Supplementary Fig. 3, Supplementary Table 6). The detailed heatmaps and tables for the categories of the “cell-to-cell signalling and interaction” and “inflammatory response” are presented in the Supplementary Figs 4–7 and Supplementary Tables 7–10

**Figure 7:**
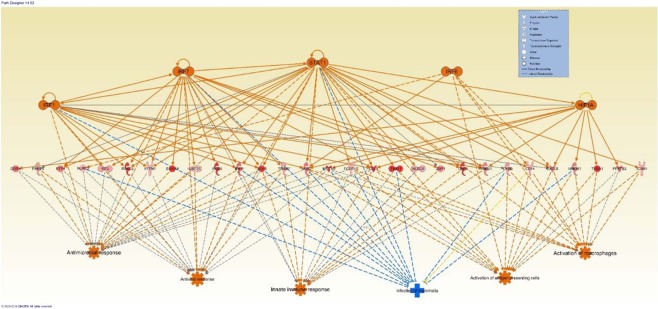
The network of transcription factors (upstream regulator, top line), overexpressed genes in high responder group at 18 hours after treatment with *Escherichia coli* (middle line), and the downstream impacts (bottom line). Orange represents activation; blue represents inhibition, yellow represent inconsistent prediction and grey indicate the absence information to predict the effect.

At the last step of the analysis, upstream regulators and downstream impacts along with DE genes were combined in IPA to predict the most probable network of the signalling pathways and biological consequences, simultaneously. The most probable network consisted of 5 transcription regulators, 29 DE genes that are directly linked to them and 6 biological consequences that are directly or indirectly linked to the DE genes and transcription factors (Fig. 7). Downstream Biological impacts that were predicted to be positively associated with the high NO^−^ responder group included: “Antimicrobial Response”, “Antiviral Response”, Innate Immune Response”, “Activation of Antigen Presenting Cells”, “Activation of Macrophages”. In addition, “Infection of Mammalia” was predicted to be negatively associated with the high responder group (Fig. 7).

## Discussion

Reductionist approaches, such as *in-vitro* models and single-cell analysis, have been instrumental in advancing the understanding of the mechanisms that control immune responses by mimicking host-pathogen interactions under experimental challenge designs^3^. Likewise, Genome-Wide Association Studies (GWAS) have been successful in detecting many Quantitative Trait Loci (QTL) that are associated with complex traits such as disease resistance^26^. However, the combination of these two approaches has recently been explored as an alternative method to investigate the genetic regulation of disease resistance. Expression QTL (eQTL) and high-throughput human *in-vitro* susceptibility testing (Hi-HOST) are just two examples of utilizing the reductionist approach in genetic studies^7,9,26,27^. Our group has recently reported notable individual variation in NO^−^ production of bovine MDMs. This cellular phenotype was strongly associated with host genetics. The heritability of NO^−^ production was 77%, similar to the heritability of NO^−^ production by peritoneal macrophages in mice^21,28^. These findings showed that NO production could be considered as a genetic index to classify MDMs. The next step was to investigate if the NO-based classification can represent any characteristics beyond the oxidative burst ability of macrophages. It has been shown previously that transcriptomic network of macrophages in conjunction with *in-silico* analysis can be employed to predict the characteristic and proinflammatory status of macrophages^24,29,30^. Therefore, in the current study, the whole transcriptome of MDMs with opposite NO-based phenotypes was compared during the response to *E. coli* to identify differentially regulated intra-cellular mechanisms and also to investigate if the DE genes be associated with *in-silico*-predicted functional characteristics of MDMs. Although analyzing the transcriptome reveals an unbiased gene expression, the results are a snapshot in time and cannot represent the dynamic regulation of macrophage functions. This limitation can be overcome by employing more than one time point and bioinformatic methods (i.e. *in-silico* prediction) to expand the snap-shot picture of gene expression in time, retrospectively (upstream regulators) and prospectively (downstream functions)^31,32^.

The DE genes were the first indicator of differentially regulated gene expression associated with NO-based classification. Among DE genes, only approximately 10% of DE genes were in common between the two time points, (Fig. 3). A similar pattern of time-dependent gene expression in bovine MDMs has been reported in response to *Mycobacterium avium* subspecies *paratuberculosis* (MAP) and *Mycobacterium bovis*^32–34^. Although the number of DE genes in the current study seems to be lower than other studies on bovine MDMs (8 to 10 times less than comparable studies), the DE genes and FC reported in the current study are the result of a comparison between two challenged groups, not a challenged group versus a control group (a common design in immunological studies)^35^. It is worth emphasizing that the only difference between the two challenged groups was their genetic background classified based on the ability to produce NO^−^. Therefore, these results indicate that genetics affects gene expression in a time-dependent manner, and it should be considered in studies on mRNA expression.

**Figure 8:**
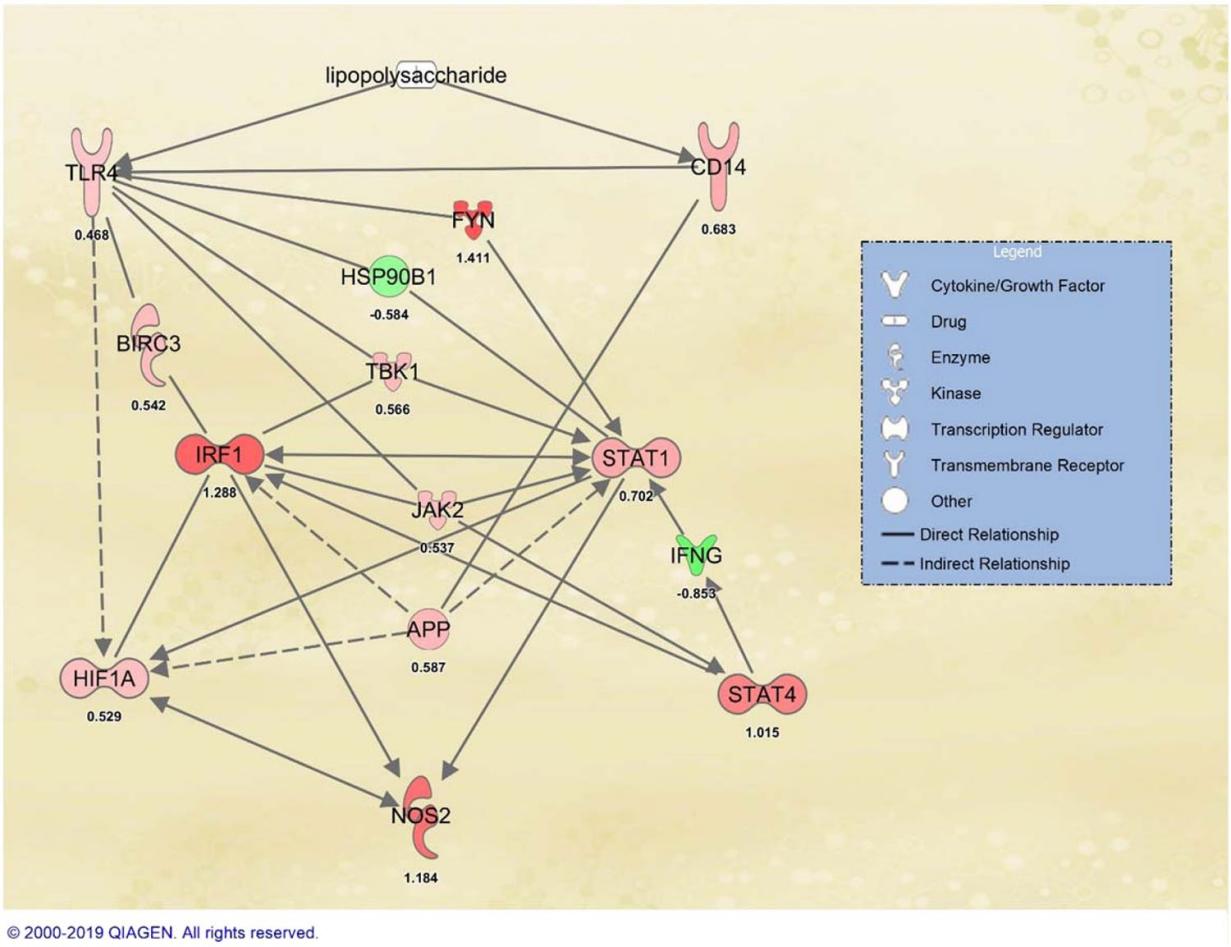
The summary of interaction between the four transcription factors that were in common between the two time points, and NOS2 (inducible nitric oxide synthase gene) and lipopolysaccharide as one of the main pathogens associated molecular pattern on *Escherichia coli.* The number under each gene symbol is the log2 fold change at 18 hours post-treatment data set. Over-expression in high responder group (or under-expression in low responder group) is represented by the density of red and under-expression in high responder group (or over-expression in low responder group) is represented by green.

Functional annotation analysis followed by *in-silico* prediction of the upstream regulator and downstream functions of DE genes revealed some key differences between high and low NO^−^ responder groups. Although some of these results are not surprising, they can indirectly indicate the absence of noise in the data set and the likelihood of accuracy of the obtained results. For instance, at both time points, LPS was found to be the most probable upstream regulator in this experiment by using an unsupervised algorithm by IPA. LPS is one the most abundant Pathogen Associated Molecular Pattern (PAMP) on the surface of Gram-negative bacteria (i.e. *E. coli*), and it perfectly aligns with the treatment that was used in this study^36^. Therefore, likely, other upstream regulators were also accurately predicted such as transcription factors. However, the other upstream regulators need to be further explored in future laboratory experiments. The *STAT1*, *STAT4*, *IRF1* and *HIF1A* that were predicted in the current study as the upstream regulators associated with NO-Based index at both 3 and 18 hours after treatment (Supplementary Table 11 and 12), are all known key transcription regulators that shape the proinflammatory response of macrophages^37–39^. In a mouse model, *IRF8*, *IRF1*, *STAT1* and *PU.1* have been shown to be a key regulator of macrophage proinflammatory and antimicrobial response by using the combination of ChIP-Seq and RNA-seq techniques. Specifically, IRF1 and STAT1 were shown to bind to the cis-regulatory region of iNOS. The NO production of macrophages was completely abrogated in *irf1*^−/−^ mouse^25^. In the current study, *IRF1*, *STAT1* and *SPI1* (gene that encodes PU.1) were differentially expressed at the 3 hours time point. In addition, *IRF8*, *IRF1*, and *STAT1* were differentially expressed at the 18 hours time point. Although it should be noted that FDR *p-value* of *IRF8* and *SPI1* was not significant in our study, their unadjusted p-values were less than 0.05. In five species of non-human primates, a conserved regulatory binding site for *STAT1*, *HIF-1*, *NFAT5* that controls the expression of iNOS has been previously reported^40^. In addition, interactions between HIF-1 and IRF-1, and HIF-1 and *STAT1* have also been reported in mice, and these regulate the expression of iNOS and induce apoptosis in cancer cells^40,41^. Although, this information supports the association between NO-based phenotypes and these TFs (summarized in Fig. 8) the genetic control in expression of these TFs seems to be less clear. Based on the design of the current study, it is possible to infer that the expressions of these TFs are genetically regulated. Although the mechanism of genetic regulation remained unanswered, it can be expected that simple genetic mechanisms such as linkage disequilibrium cannot explain the variation in their expression. These TFs are located on 3 different chromosomes on the bovine genome (*STAT1* and *STAT4* on Bovine Autosomal Chromosome (BTA)-2, *IRF1* on the BTA-7 and *HIF1A* on the BTA-10) and it is unlikely to expect that their expressions are controlled via a physical linkage. Whether there is one mutation in one regulatory molecule upstream of all these TFs or another mechanism that links and regulates the expression of these TFs needs to be further investigated. The presence of two clusters among differentially regulated TFs should also be noted. Markedly, the cluster that contains TF with role in response hypoxia (*HIF1A*) is growing during the time (Fig. 6). The expression of HIF1A after stimulation of macrophage with LPS has been previously documented. The role of HIF1A in regulation of metabolic process and pro-inflammatory characteristics of macrophages is also known^42,43^. However, the results of the current study suggest a genetic regulation mechanism on activation of HIF1A. In addition, due to the role of HIF1A in trained immunity^44^, the current study indicates the possibility of genetic effect on trained immunity in outbred populations. This hypothesis needs further investigation on identifying genetic markers that are associated with trained immunity, macrophage polarization, response to hypoxic and oxidative stress in macrophages.

Among enriched canonical pathways at 3 hours time point, “Fcγ Receptor-mediated Phagocytosis in Macrophages and Monocytes” is notable. We have previously shown a strong and significant correlation between NO^−^ production and phagocytosis in bovine MDMs in response to *E. coli* and *S. aureus*^21^. The VEGF signalling pathway, enriched at 3 hours, regulates angiogenesis and lymphangiogenesis in the host during inflammation. The association between VEGF pathway and macrophages NO^−^ production has been previously reported in mice and plays a vital role in antigen clearance and regulation of inflammation^45–47^. Similarly, the PI3k-dependent pathways have different roles in the cells of the immune system from the regulation of metabolism to down-regulation of inflammation and macrophage polarization^48,49^. Inhibition of this pathway has resulted in a reduction of proinflammatory cytokine expression in response to LPS in THP-1 derived macrophages^50^.

The enriched pathways at 18 hours are either directly related to proinflammatory responses (i.e. *STAT3*^51^, iNOS signalling, Inflammasome pathways^52^ and IL-2 Signalling^53^ or they show inflammatory status in a tissue or an organ (i.e. Neuroinflammatory Signalling pathway) (Fig. 5). The “Production of Nitric Oxide and Reactive Oxygen Species in Macrophages” pathway had the lowest p-value and the “Inflammasome Pathway” was the most enriched pathway (20%) among all pathways. Although it is not surprising to find nitric oxide pathway is enriched in this data set, it indicates the accuracy of the methods that were employed in the current study, from the wet-lab (cell culture, stimulation and measuring the NO response) to the bioinformatic analysis (trimming and aligning the reads, quantifying the expression level and thresholds for DE genes). The most enriched pathways at 18 hours timepoint (“Inflammasome Pathway”) is also a known pathway to induce NO^−^ production in macrophages^52^. The epigenetic mechanism of interactions between inflammasome, PARP-1 and iNOS have recently been reported in mice^54^. The inflammasome pathway that is enriched based on DE genes in the current study, its association with the level of NO^−^ production and the stimulator that was used here (*E. coli*), align with this recent discovery.

Combining of all these pathways resembles a distinct *in-silico*-predicted proinflammatory profile between high and low NO^−^ responder groups as it is illustrated in Supplementary Figs. 6 and 7 and Supplementary Table 9 and 10. The “Inflammatory Response”, “Cell-to-Cell Signaling”, “Cellular Movement” and “Immune Cell Trafficking” were predicted to be significantly associated with the high responder phenotype at both time points (Supplementary Table 5–10. These pathways have also been reported to be enriched in bovine MDM in response to MAP, indicating their importance in macrophage responses^32^. These *in-silico*-predicted functions can constitute a distinct immunological response such as stronger antimicrobial and antiviral responses, a higher level of antigen presentation in the high NO^−^ responder groups which can lead to stronger innate responses and reduced infection (Fig. 7). Since these biological impacts of DE genes are predicted based on previously published literature, these predicted functions need to be validated in future studies using *in-vivo* experiments.

It is also worth looking at the results of the current study from the lens of the macrophage polarization paradigm. Macrophages are known to be polarized with distinct characteristics in a continuum of phenotypes from M1 (pro-inflammatory) to M2 (anti-inflammatory), with many stages in between^22–24^. This polarization is classified based on the stimulatory signals that macrophages receive, but this concept has been mainly generated from studies using inbred mice models. In the current study, both phenotypes (low and high responders) received the same stimulatory signal (GM-CSF and *E. coli* under the same environmental condition), but their functional characteristics are predicted to be distinct. Therefore, genetics can add a second dimension to the linear continuum of the macrophage polarization model. The expression of M1-associated genes such as *STAT1* (FC = 1.62, FDR = 0.002), *IRF1* (FC = 2.44, FDR = 2.83e-11), *HIF-1A* (FC = 1.44, FDR = 0.046), *IL8* (FC = 2.2, FDR = 1.48e-4), *CCL5* (FC = 5.83, FDR = 0.04), *iNOS* (FC = 2.26, FDR = 1.89e-4), *CD38* (FC = 1.91, FDR = 0.023) and *CD14* (FC = 1.60, FDR = 0.005) were found to be significantly more expressed in this high responder group^22–24,55,56^. Whereas, the expression of M2-associated genes such as *MYC* (FC = −1.83, FDR = 0.031), *GPNMB* (FC = −2.0, FDR = 4.34e-4), *MSR1* (FC = −1.85, FRD = 0.010), *DHCR24* (FC = −1.79, FDR = 0.016) and *LGMN* (FC = −2.86, FDR = 1.14e-9) are more expressed in low NO^−^ responders^18,57–60^. It should also be noted that the expression of *GATA3* and *IRF4*, known M2-associated TFs^23^, were predicted to be inhibited in the high responder group (or activated in low responder group) based on DE genes at 18 hours (Supplementary Table 4). Based on these results, there is a notable overlap between NO-based classification of bovine MDM in the current study and M1/2 macrophage polarization in mice or humans. These results indicate that stimulatory signals are not the sole determinant of macrophages polarization, and the phenotype is shaped in the interaction between genetic and stimulatory signals (also known as gene by environment effects). The gene by environment effects might be able to explain some individual differences in response to pathogens. Casey *et.al.* have shown the transcriptomic changes of bovine MDMs in response to MAP at 2 and 6 hours after challenge^32^. At FDR level of less than 0.1, 36 and 85 genes are in common between the study by Casey *et.al.* and the 3 hours time point of the current study^32^. Among these common genes, 10 genes with direct role on innate immune responses should be noted (TNFAIP3, TRAF3, TNIP1, BIRC3, CHSTS3, IRF1, NOS2, PELI1, PTGS2 and SLC25A19)^32^. This overlap represents the possibility of further investigation on the genetic regulation of Johne’s disease (caused by MAP in cattle) using the *in-vitro* model and NO-based classification.

In conclusion, the results of this study indicate a distinct proinflammatory profile between MDMs that are classified based on NO^−^ production. It is also predicted that cattle that are classified as the high responder will likely mount a stronger innate response and have a lower incidence of infectious disease when NO^−^ is required to help control infection. Moreover, this genetically-depended distinct proinflammatory response might be able to describe the individual differences in the progress of some infectious diseases that are linked to inflammatory responses, such as Johne’s disease in cattle or Crohn’s disease in human. A notable difference in the progress of these diseases has been reported to be associated with the inflammatory response of the host. Therefore, the NO-based classification might be able to provide a platform to investigate the genetic mechanism(s) that shapes the outcome of host infection.

## Material and Methods

### Monocytes-Derived Macrophages and *E. coli* Stimulation

Blood samples were collected from the tail vein of 6 Holstein mid-lactating cows from the research herd at the University of Guelph. Based on our previous study^21^, these samples were divided into high (n = 3) and low (n = 3) responder groups based on *in-vitro* NO^−^ response to *E. coli*. The average of NO^−^ response 48 hours after the challenge was 13.1 µM (Standard Error: 0.66) and 5.3 µM (Standard Error: 0.47) for high and low responder groups, respectively (*p-value* of T-test between high and low responder groups was < 0.002) (Supplementary Fig. 8). The MDMs were generated in a serum-free culture system by the method previously published^21^. Briefly, blood Mononuclear Cells (BMCs) were purified based on the gradient centrifuge separation method and cultured for 2 hours in Monocyte Attachment Medium (PromoCell, Heidelberg, Germany) at 37 °C. Non-adherent cells were removed and the medium was replaced with AIM V® Medium (Thermo Fisher Scientific Inc., Mississauga, ON) in the presence of 5 ng/ml recombinant bovine Granulocyte-macrophage colony-stimulating factor (GM-CSF, Kingfisher Biotech, St. Paul, MN) and 5% CO2. After six days of incubation, the culture flasks were vigorously washed to remove any dead cells before the harvest. Samples were inspected visually under the microscope to confirm cell morphology before harvesting. Adherent cells were detached from the flask using TrypLE™ Select Enzyme (Thermo Fisher Scientific Inc., Mississauga, ON). After harvesting, all samples were tested for uniformity of the cell population and cell dimensions using S type cassettes in Moxi Z automated cell counter (ORFLO Technologies, ID, USA). All samples used in this study had the Moxi Population Index (MPI) of above 0.9 and average diameter of 14 µM. Phenotypic characteristics of macrophages (strong auto-fluorescence, CD14^+^, CD205^−^) were analyzed using flow cytometry to determine the proportion of macrophages among the harvested cells^61–64^. Harvested cells were labelled with RPE conjugated mouse anti-bovine CD205 (Clone: IL-A114), to check the presence of monocyte-derived dendritic cells and ALEXA FLUOR^®^ 647 conjugated mouse anti-human CD14 (Clone: TÜK4) to check the presence of myeloid cells, separately. The labelled samples were analyzed using the BD Accuri C6 flow cytometer (Becton Dickinson, Franklin Lakes, NJ) and the data from the flow cytometer were analyzed by FlowJo V.10 (FlowJo LLC, Ashland, OR). The fluorescence emission of an unlabeled harvested cells was compared with the emission of unlabeled fresh BMCs in 533/30 filter excited by the blue laser to test the auto-fluorescence as an indicator for macrophages (Supplementary Fig. 9). MDMs from each sample were seeded in 4 wells in two 24-well plates in AIM V® at the concentration of 0.4 × 10^6^ cells per well. Each plate was assigned to one time-point. One well of each sample was assigned as the control and the other well was exposed to *E. coli* (MOI: 5).

All the procedure and handling of the animals were approved by the animal care committee of the University of Guelph. All experiments were performed in accordance with relevant guidelines and regulations.

### RNA isolation, library construction and Sequencing

At 3 and 18 hours after treatment, total RNA was extracted from all four wells, using TRIzol Reagent (Thermo Fisher Scientific Inc., Mississauga, ON) according to the manufacturer’s protocol. Briefly, 1 ml of Trizol Reagent was used to extract total RNA from 0.4 × 10^6^ MDMs based on the company’s recommendation. The extracted samples were treated with DNase to remove any possible DNA contamination. The quantity of the purified RNA samples was measured by the RNA High Sensitivity kit in the Qubit Fluorometric Quantification system (Thermo Fisher Scientific Inc., Mississauga, ON) and the qualities were checked by the 2100 Bioanalyzer (Agilent, Santa Clara, CA). The average RNA integrity was 9.3 (standard deviation = 0.50), indicating good RNA quality^65^.

Library construction was performed using the TruSeq Stranded mRNA Libraries Prep kit (Illumina Inc. San Diego, CA). Adapters were ligated to the ends of double-stranded cDNA and PCR amplified to create libraries^65,66^. An equal amount of each library was pooled together and was paired-end sequenced in HiSeq-4000 system (Illumina Inc. San Diego, CA) to generate 150 bp reads.

## Bioinformatics Analysis

### Differentially expressed genes

Sequencing reads were filtered for quality and removing the index (universal Illumina index) using Trimmomatic in the pair-end mode^67^. Nucleotides with quality of less than 30 (in Phred score) at the beginning and at the end of the reads were removed^65,68^. In addition, reads shorter than 100 bp or with a quality of less than 20 (in Phred score) in 5 adjacent base pairs were removed from the analysis. The quality of the reads was checked by FastQC (Babraham Bioinformatics, v. 0.11.5) before and after trimming. At the next step, clean reads were mapped to the bovine reference genome (UMD 3.1, Release 94) by using Spliced Transcripts Alignment to a Reference (STAR) v. 2.7.0a^69,70^. The quantity of expression per annotated genes was calculated by using RNA-Seq by Expectation Maximization (RSEM) package (v. 1.3)^71^. The raw count data were imported by R’ Bioconductor package “tximport” (v 1.10.1) and analyzed by DESeq2 (v. 1.22.2) by employing negative binomial GLM fitting approach^72,73^. Every sample was numbered within a phenotype and consisted of two reads files, treated and control. The model term in DESeq2 was designed to calculate the differential expression in fold change (FC) between the phenotypes in the treated group after accounting for the expression level of the respective control sample. To correct for multiple comparison error, *p-values* for each gene was adjusted based on the Benjamini and Hochberg method^74^. Genes with the absolute FC of greater than 1.5 and adjusted *p-value* of less than 0.1 were considered Differentially Expressed (DE).

### Functional analysis

The output of DESeq2 analysis was imported to Ingenuity Pathway Analysis (IPA) cloudware (QIAGEN, Redwood City, www.qiagen.com/ingenuity), to identify Gene Ontology (GO) terms, metabolic pathways, upstream regulators and functional networks among the DE genes^75^. The “core analysis” function in IPA was used as suggested by the manual with following criteria: removing genes with an ambiguated identifier (unmapped), absolute log2 FC of less than 0.58, and *q-value* of more than 0.1. The Fisher Exact test followed by adjusting p-value based on the Benjamini and Hochberg method was used by the IPA to identify statistically significant enriched metabolic, pathways and functional networks. The interactions between the DE genes were investigated using the Search Tool for Retrieval of Interacting Genes/Proteins (STRING, v. 11)^76^.

## Supplementary information

Supplementary Information 1

Supplementary Information 2

## Data Availability

The datasets generated and analyzed during the current study are available in the Gene Expression Omnibus repository, via accession code GSE136722.
