# Supplementary material for: Transcriptomic Profiles of Monocyte-Derived Macrophages in Response to Escherichia coli is Associated with the Host Genetics
Source: Sci Rep. 2020 Jan 14;10:271. doi: 10.1038/s41598-019-57089-0 (PMC6959288; doi:10.1038/s41598-019-57089-0)
Supplement: Supplementary file 1 — Supplementary Information 1 [file 41598_2019_57089_MOESM1_ESM.docx]

**Supplementary Figures**

**Transcriptomic Profiles of Monocyte-Derived Macrophages in Response to *Escherichia coli* is Associated with the Host Genetics**

Mehdi Emam^1,2^, Angela Cánovas^2^, Alma D. Islas-Trejo^3^, Pablo A.S. Fonseca^2^, Juan. F. Medrano^3^ & Bonnie Mallard^1,2^

^1^Department of Pathobiology, Ontario Veterinary College, University of Guelph, Guelph, Ontario

^2^Centre for Genetic Improvement of Livestock, Department of Animal Biosciences, Ontario Agricultural College, University of Guelph, Guelph, Ontario

^3^Department of Animal Science, University of California-Davis, Davis, California

**Corresponding Author: Mehdi Emam**

[**semam@uoguelph.ca**](mailto:semam@uoguelph.ca)

419 Gordon St, Bldg #89, Guelph, ON N1G 2W1

semam@uoguelph.ca

519 824 4120 x54761


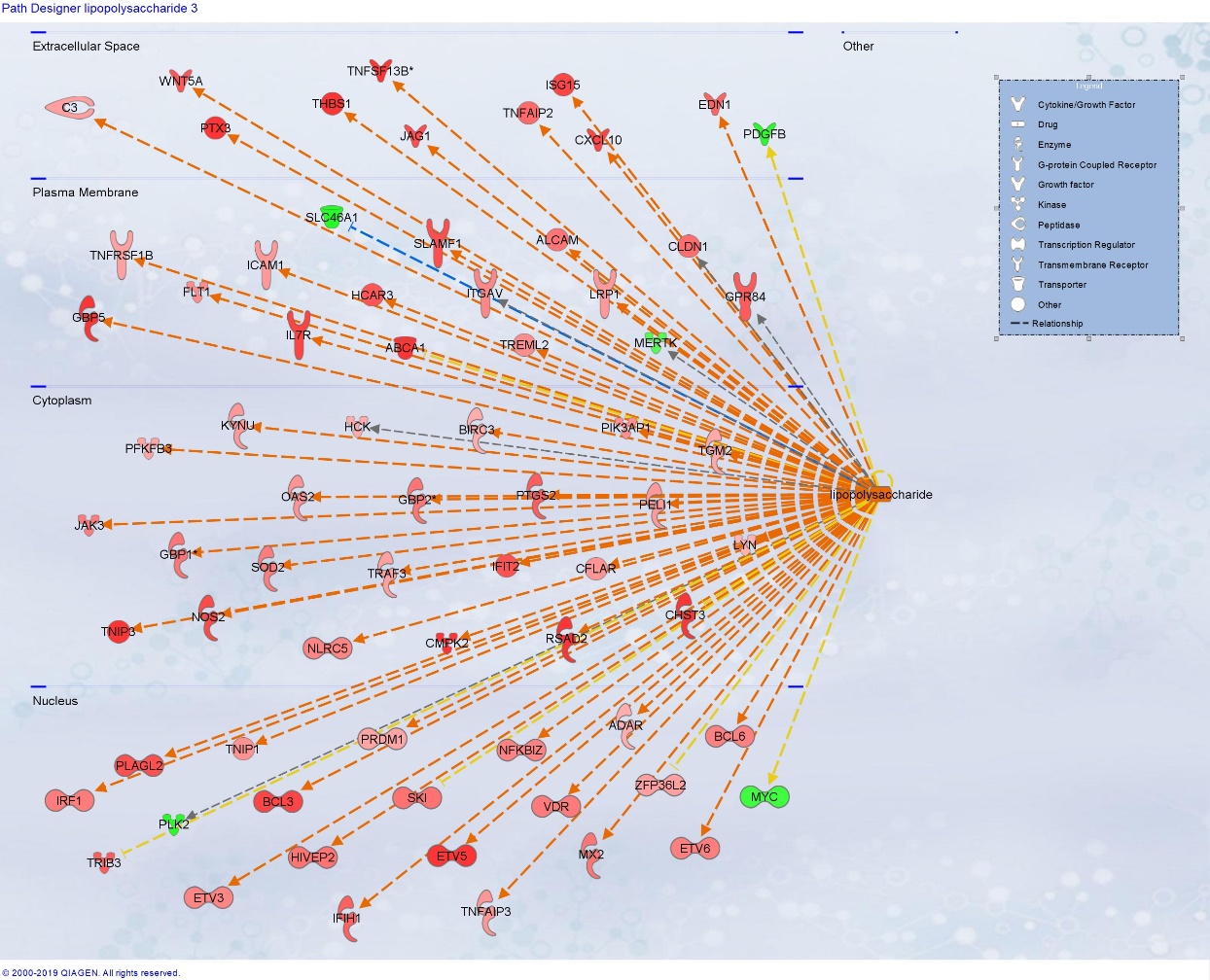


**Supplementary Figure 1** - Differentially expressed genes in monocyte-derived macrophage at 3 hours after treatment with *Escherichia coli,* which are known to be affected by lipopolysaccharide. Over-expression in the high responder group (or under-expression in low responder group) is represented by the density of red and under-expression in the high responder group (or over-expression in the low responder group) is represented by green. Orange lines represent activation; blue represents inhibition, yellow represents inconsistent prediction.


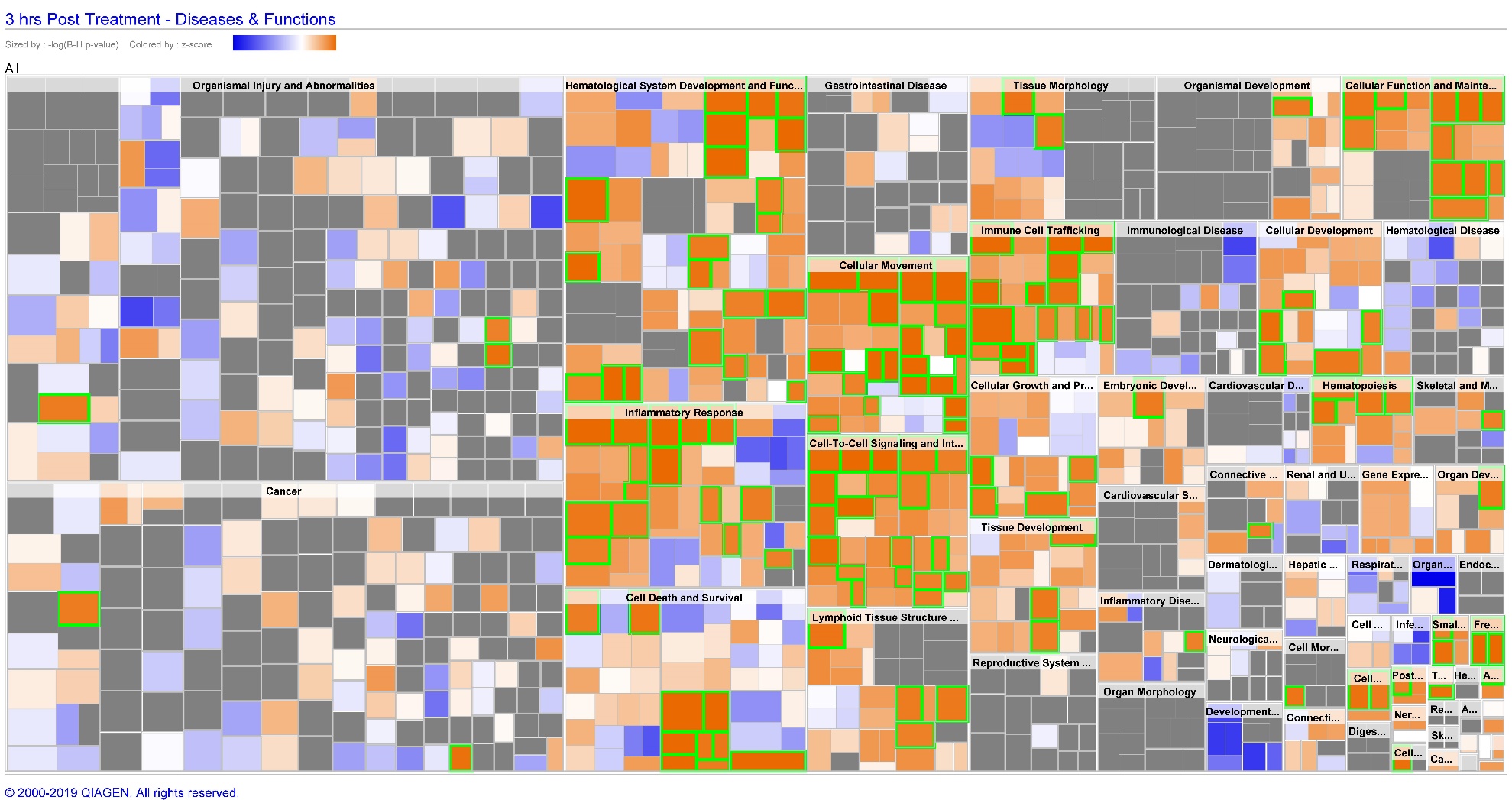


**Supplementary Figure 2** – Overview of diseases and biological functions that are predicted to be associated with high responder group based on differentially expressed genes at 3 hours time point. The size of each square represents the -log10 of the predicted function/disease (all predicted functions/diseases are under 0.05 FRD p-value). The density of orange represents the activation z-score and blue represents the inhibition z-score; white represents the z score of zero and grey represents no prediction value. Four sides of squares with absolute z-sore of greater than two are highlighted in green.


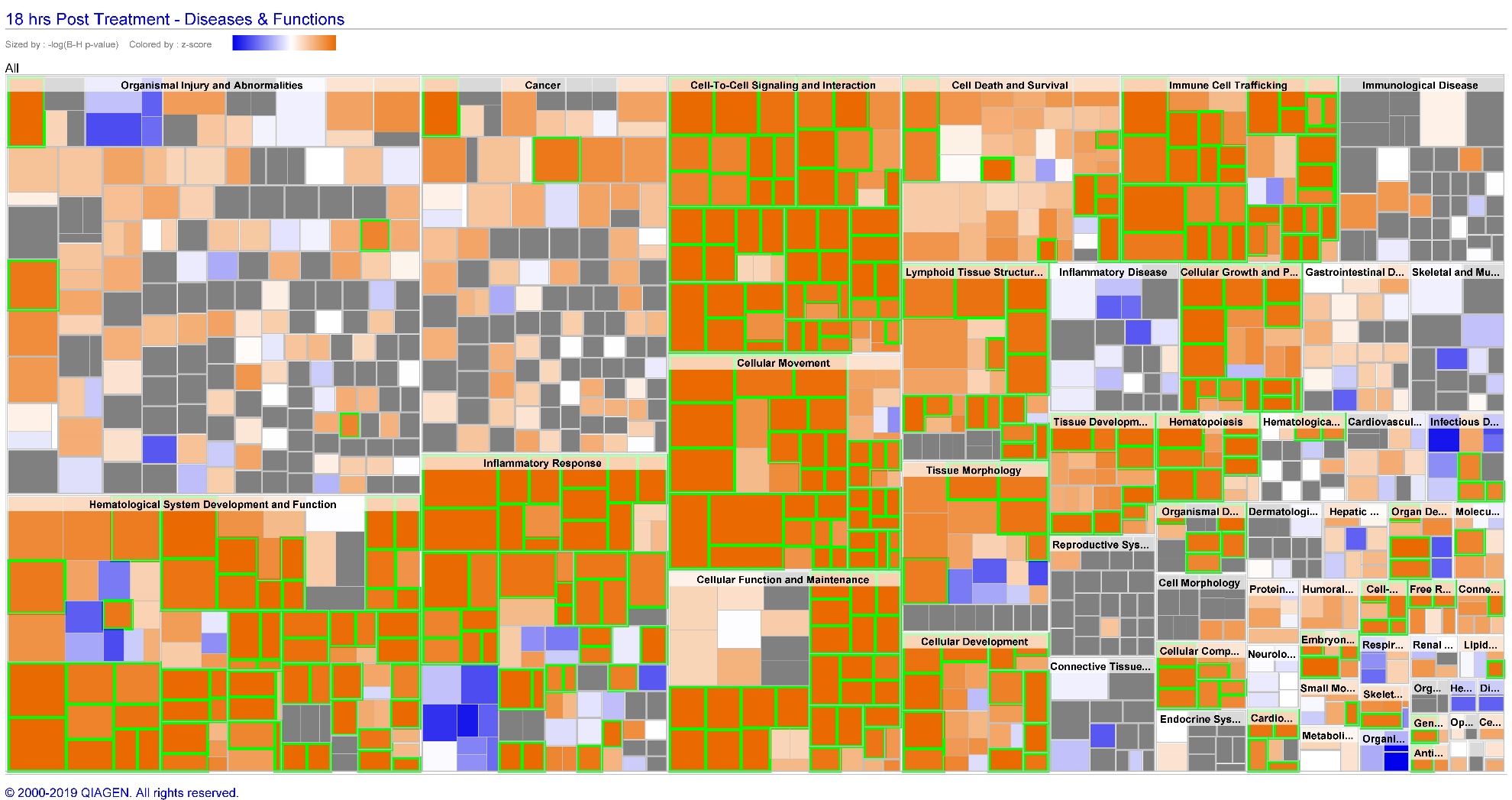


**Supplementary Figure 3** – Overview of diseases and biological functions that are predicted to be associated with the high responder group based on differentially expressed genes at 18 hours time point. The size of each square represents the -log10 of the predicted function/disease (all predicted functions/diseases are under 0.05 FRD p-value). The density of orange represents the activation z-score and blue represents the inhibition z-score, white represents z score of zero and grey represents no prediction value. Four sides of squares with absolute z-sore of greater than two are highlighted in green.


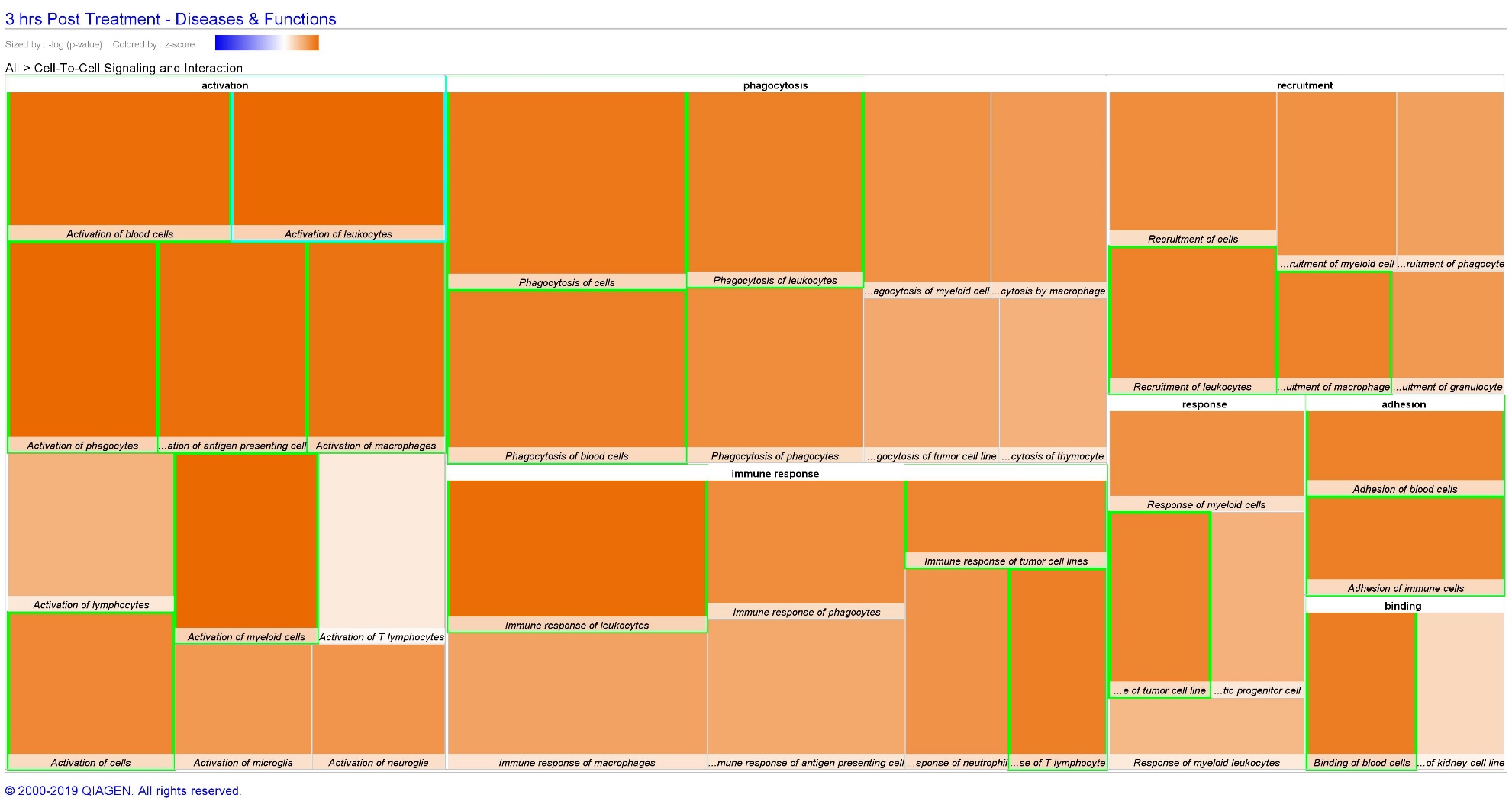


**Supplementary Figure 4** – “Cell-to-Cell Signaling and Interaction” category of diseases and biological functions that are predicted to be associated with the high responder group based on differentially expressed genes at 3 hours time point. The size of each square represents the -log10 of the predicted function/disease (all predicted functions/diseases are under 0.05 FRD p-value). The density of orange represents the activation z-score and blue represents the inhibition z-score, white represents z score of zero. Four sides of squares with absolute z-sore of greater than 2 are highlighted in green.


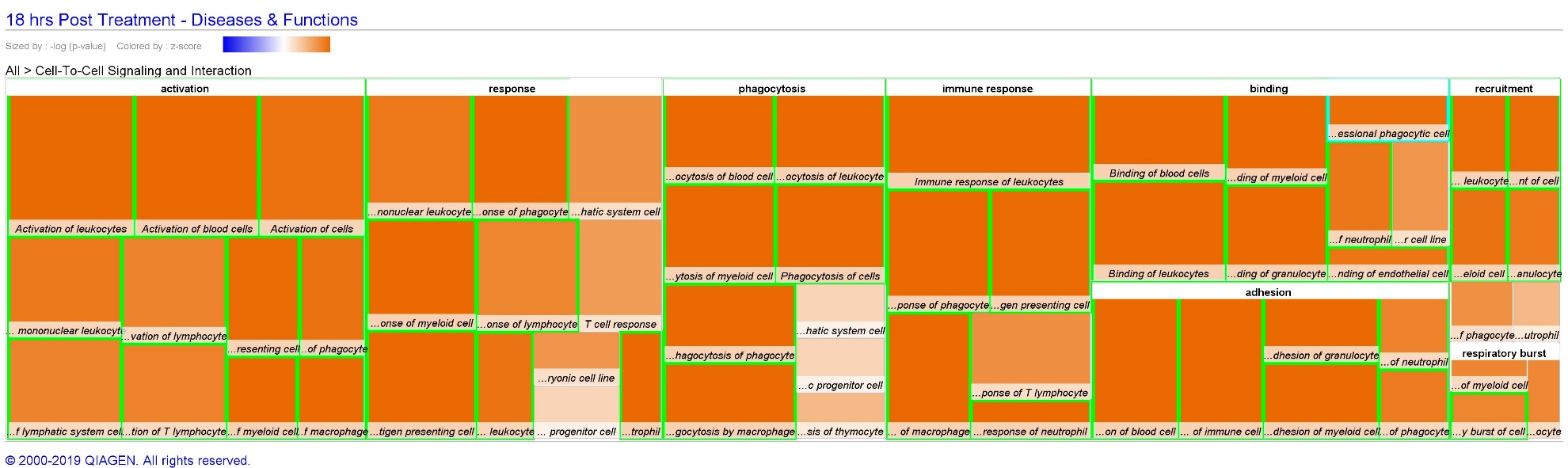


**Supplementary Figure 5** – “Cell-to-Cell Signaling and Interaction” category of diseases and biological functions that are predicted to be associated with the high responder group based on differentially expressed genes at 3 hours time point. The size of each square represents the -log10 of the predicted function/disease (all predicted functions/diseases are under 0.05 FRD p-value). The density of orange represent the activation z-score, and blue represents the inhibition z-score, white represents z score of zero. Four sides of squares with absolute z-sore of greater than two are highlighted in green.


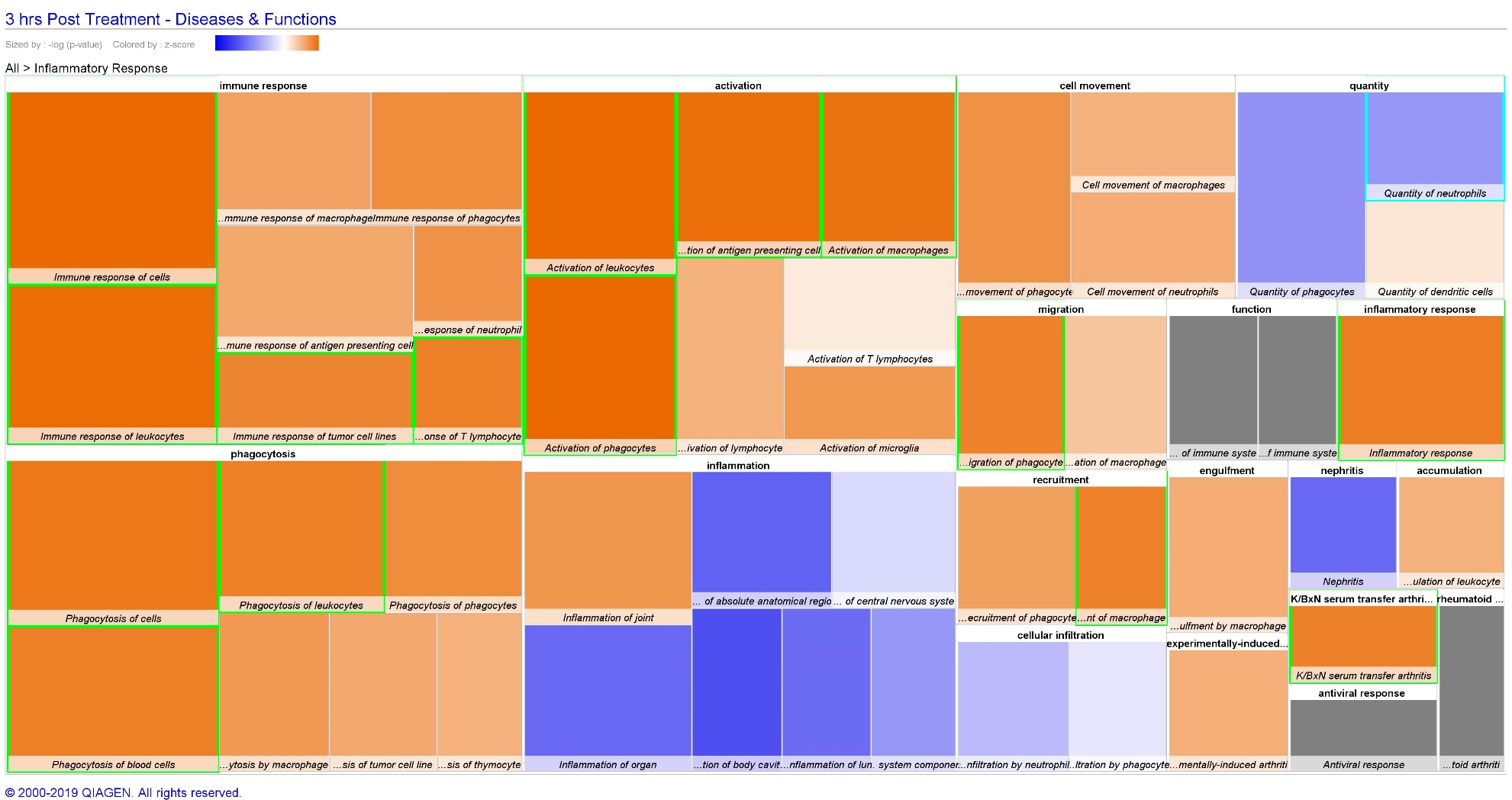


**Supplementary Figure 6** – “Inflammatory Response” category of diseases and biological functions that are predicted to be associated with the high responder group based on differentially expressed genes at 3 hours time point. The size of each square represents the -log10 of the predicted function/disease (all predicted functions/diseases are under 0.05 FRD p-value). The density of orange represent the activation z-score, and blue represents the inhibition z-score, white represents z score of zero. Four sides of squares with absolute z-sore of greater than two are highlighted in green.


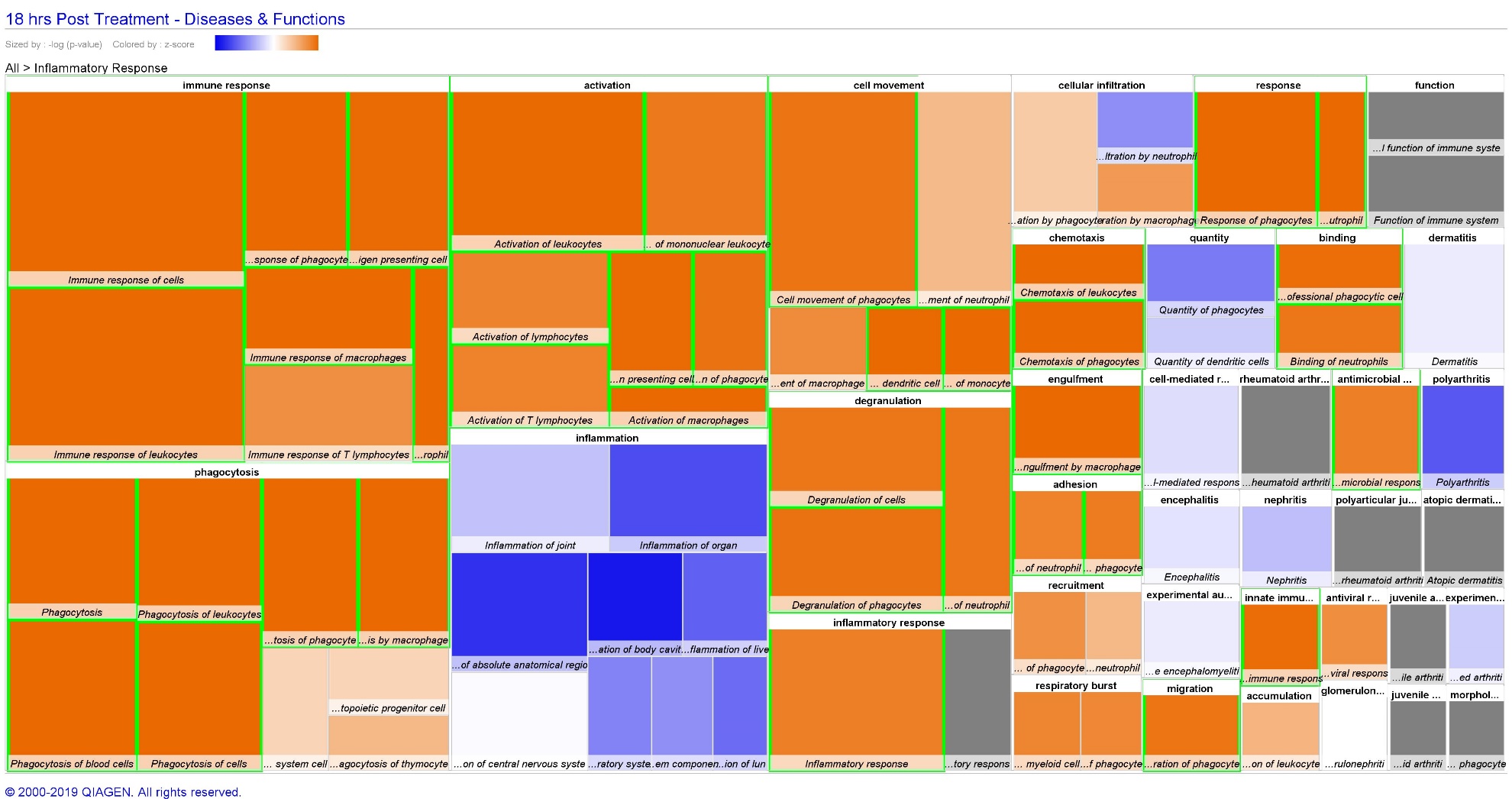


**Supplementary Figure 7** – “Inflammatory Response” category of diseases and biological functions that are predicted to be associated with the high responder group based on differentially expressed genes at 18 hours time point. The size of each square represents the -log10 of the predicted function/disease (all predicted functions/diseases are under 0.05 FRD p-value). The density of orange represent the activation z-score, and blue represents the inhibition z-score, white represents the z score of zero. Four sides of squares with absolute z-sore of greater than two are highlighted in green.

**
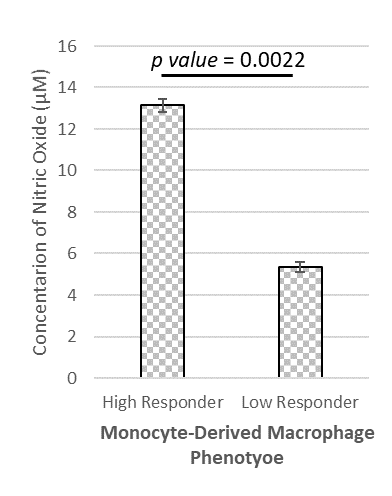
**

**Supplementary Figure 8-** The concentration of Nitric Oxide (NO^-^) in supernatant bovine Monocyte-Derived Macrophage (MDM) culture after 48 hours exposure to *Escherichia coli***.** MDMs were exposed to inactivated *E. coli* (MOI: 5) for 48 hours. The supernatant was collected and the concentration of NO^-^ was measured with the Measure-iT™ High-Sensitivity Nitrite Assay Kit (Thermo Fisher Scientific Inc., Mississauga, ON). The concentration of NO- for every sample was subtracted by its replicate in the control group (untreated).

**
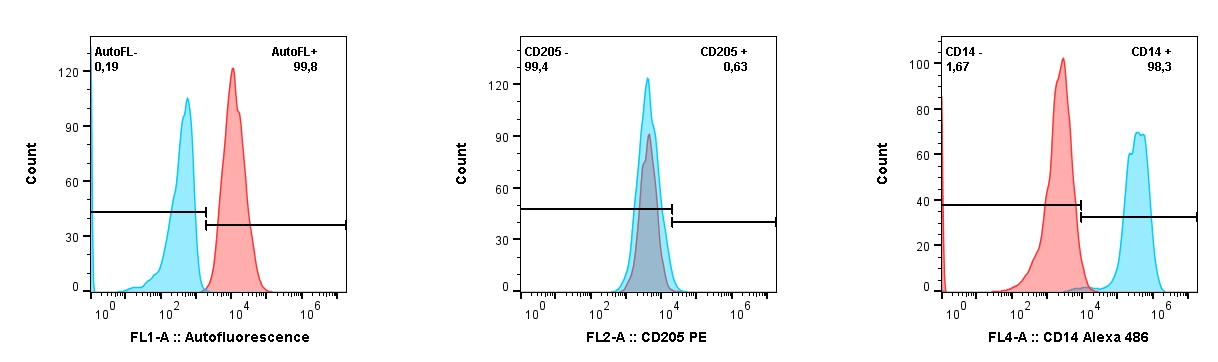
**

**Supplementary Figure 9 -** Phenotypic characteristics of harvested cells after six days of *in-vitro* incubation in serum-free media supplemented with recombinant bovine Granulocyte-macrophage colony-stimulating factor. The harvested cells were stained with Alexa Flour 647 conjugated anti-human CD-14 (Clone TÜK4), and phytoerythrin (PE) conjugated anti-bovine CD-205, separately. The cells were analyzed in BD Accuri™ C6 cytometer against unstained harvested cells and one unstained blood mononuclear cells as the reference for autofluorescence in 533/30 filter excited by the blue laser. The data from the flow cytometer were analyzed and graphed using FlowJo (v. 10).
